# Bacterial diversity of bat guano from Cabalyorisa Cave, Mabini, Pangasinan, Philippines: A first report on the metagenome of Philippine bat guano

**DOI:** 10.1371/journal.pone.0200095

**Published:** 2018-07-19

**Authors:** Marian P. De Leon, Andrew D. Montecillo, Dale S. Pinili, Maria Auxilia T. Siringan, Doo-Sang Park

**Affiliations:** 1 Microbial Culture Collection, Museum of Natural History, University of the Philippines Los Baños, College, Laguna, Philippines; 2 Microbiology Division, Institute of Biological Sciences, College of Arts and Sciences, University of the Philippines Los Baños, College, Laguna, Philippines; 3 Plant Breeding, Genetics and Biotechnology Division, International Rice Research Institute, Los Baños, Laguna, Philippines; 4 Microbiological Research and Services Laboratory, Natural Sciences Research Institute, University of the Philippines Diliman, Quezon City, Philippines; 5 Korean Collection for Type Cultures, Biological Resource Center, Korea Research Institute of Bioscience and Biotechnology, Jeongeup, South Korea; University of Pretoria, SOUTH AFRICA

## Abstract

Bats are highly diverse and ecologically valuable mammals. They serve as host to bacteria, viruses and fungi that are either beneficial or harmful to its colony as well as to other groups of cave organisms. The bacterial diversity of two bat guano samples, C1 and C2, from Cabalyorisa Cave, Mabini, Pangasinan, Philippines were investigated using 16S rRNA gene amplicon sequencing. V3-V4 hypervariable regions were amplified and then sequenced using Illumina MiSeq 250 PE system. Reads were processed using Mothur and QIIME pipelines and assigned 12,345 OTUs for C1 and 5,408 OTUs for C2. The most dominant OTUs in C1 belong to the Proteobacteria (61.7%), Actinobacteria (19.4%), Bacteroidetes (4.2%), Firmicutes (2.7%), Chloroflexi (2.5%), candidate phylum TM7 (2.3%) and Planctomycetes (1.9%) while Proteobacteria (61.7%) and Actinobacteria (34.9%) dominated C2. Large proportion of sequence reads mainly associated with unclassified bacteria indicated possible occurrence of novel bacteria in both samples. XRF spectrophotometric analyses of C1 and C2 guano revealed significant differences in the composition of both major and trace elements. C1 guano recorded high levels of Si, Fe, Mg, Al, Mn, Ti and Cu while C2 samples registered high concentrations of Ca, P, S, Zn and Cr. Community structure of the samples were compared with other published community profiling studies from Finland (SRR868695), Meghalaya, Northeast India (SRR1793374) and Maharashtra State, India (CGS). Core microbiome among samples were determined for comparison. Variations were observed among previously studied guano samples and the Cabalyorisa Cave samples were attributed to either bat sources or age of the guano. This is the first study on bacterial diversity of guano in the Philippines through high-throughput sequencing.

## Introduction

Bats (Order: Chiroptera) are highly diverse and ecologically valuable comprising 25% of living mammalian species. The most abundant group of mammals based on the number of individuals, they evolved into an incredibly rich diversity that roost in foliages, caves, rock crevices even in man-made structures[1, 2]. They feed on insects, nectar, fruits, seeds, fish, frogs and small mammals [3, 4].

Bats serve the ecosystem as pollinators, agents of seed dispersal and sources of guano-based fertilizers. As biological agents to control pest population, they limit spread of human diseases and prevent significant economic losses on crops and livestock [4–7]. Through the production of guano, their accumulated excrement, bats are also responsible for nutrient cycling. As the main source of energy for a wide range of unique species found on specific caves, guano deposits form the basis of cave ecosystems [7]. The formation of bat guano is an interesting interplay among bats as primary producers, the nutrients present in the food they eat and the biological and environmental changes inside the cave ecosystem. Guano is noted for its agricultural benefits as an ideal fertilizer attributed to its richness in carbon, nitrogen, potassium and phosphorus. In the US, Asia, Cuba and South America, guano is being marketed as the best organic fertilizer available. In the Philippines, caves in the Bicol Region, Pangasinan and Samal (Davao del Norte) are few places that produce abundant guano in high risk of disturbance due to illegal extraction. According to local government officials, guano-mining is inevitable since it is the source of income among the locals and, likewise, a free organic fertilizer for agricultural crops within the vicinity of the caves.

As prey and predator, bats have been implicated in epidemiologic cycles of several emerging and re-emerging zoonoses [8] and carriers of pathogenic agents that include more than 200 different types of viruses such as rabies [9], Ebola and Marburg viruses [10–12], and Severe Acute Respiratory Syndrome (SARS) coronavirus [5, 13, 14]. Fungi like *Histoplasma capsulatum* [15–18], *Geomyces destructans* [19] and *Pseudogymnoascus destructans* [20] were reported to be isolated, considered as cause of infection and death in hibernating bats. Although bacterial colonization has been observed only on samples of rocks, cave wall and/or ceiling paintings, dripping waters, springs and underwater passages, oligotrophic cave-dwelling microbial species have been described as phylogenetically diverse with lineages across the breadth of the bacteria. Presence of pathogenic enteric bacteria mainly from the family Enterobacteriaceae and some bacterial pathogens common in human and animal diseases (e.g. *Pasteurella*, *Salmonella*, *Escherichia* and *Yersinia* spp.) [21, 22] was reported to be affected by the foraging habits, diet and activities of the bats inside and outside of caves. Other bacterial pathogens (e.g. *Bartonella*, *Borrelia*, *Leptospira* spp., *Serratia*, *Pseudomonas*, *Enterobacter*, *Acinetobacter*, *Bacillus*, *Arthrobacter* and *Micrococcus* spp.) provided evidence for novel species that seem to be specific for bat hosts with probable medical importance to humans and other animals [23–26]. These harmful microorganisms may also contribute to continuous decline of bat population in caves. In addition, human-induced environmental stresses such as habitat destruction and fragmentation, overhunting for bush meat, and increased use of pesticides also have negative impact to bat population in caves [4]. Thus a persistent call for global bat conservation is necessary.

So far, the discovery of novel and potentially pathogenic bacteria and its interaction with the environment is limited by culture-based approaches. Culture-independent methods are essential to understand the genetic diversity, population structure, and ecological roles of the majority of microorganisms [27]. High-throughput screening allows the paradigm shift on microbiology and bioinformatics towards modern metagenomics that augment the limitations of culture-based methods [28, 29]. Availability and accessibility of cost-effective next-generation high-throughput sequencing methods improved understanding of the assembly, evolution and functions of ecological communities. Metagenomic studies using 16S rRNA gene sequencing were used to characterize gut and guano microbiome of several bat species [22, 27, 30–32] and feces of different animals to determine their diet as well as the diversity of metabolic functional genes (i.e. enzymes) [33–35].

To date, there have been no reports on the bacterial diversity of guano from Philippine caves. There is limited local information in terms of benefits and harmful effects as well as ecological importance. Bacterial diversity of guano may give insights on its potential as source of nutrients for plant growth and their impacts on human health. This is the first report on the bacterial diversity of bat guano using metagenomics in the Philippines particularly at Cabalyorisa Cave, Mabini, Pangasinan.

## Materials and methods

### Sample collection

Guano samples were collected from two large chambers, Chamber 1 (C1) and Chamber 2 (C2) in Cabalyorisa Cave Complex, Mabini, Pangasinan, Philippines (N16°00'22.6"E and N119°56'35.2"E, respectively at 100 msl). Prior to sampling, a Wildlife Gratuitous Permit (WGP) (2015–002) has been granted by the Department of Environment and Natural Resources (DENR), La Union, Philippines. The WGP allowed the conduct of field collection for the assessment of bat guano community in selected caves in Mabini, Pangasinan, Philippines with clearances and endorsements of the local government unit.

The first guano sample, C1, was collected from a chamber located 50 meters from the cave entrance. Patches and pool of powdery guano were collected in between rocks, rock crevices and on pot holes of the breakdown pile of about 3–4.5 m high and 3–5 m from the ceiling using sterile hand trowel. The chamber was about 9 m at its widest and near a pool of water. The second sample, C2, was collected 77 m further up near the flyway of bats. It is 6 m at its widest with ceiling of 3 to 7 m high [36] and host sizable roost of bats of about 10–100 bat individuals. This chamber is an important flyway for bats roosting further in the cave and host about 1–10 bat individuals. The floor of the site was uneven, made of large boulders and rock formations that holds pool of guano. Guano were collected from seven sites in each chamber, pooled and placed inside re-sealable zipper plastic bags using sterile shovel. Composite samples were then brought to the laboratory inside an ice chest and subjected to immediate DNA extraction and X-ray Fluorescence (XRF) spectrophotometric analysis.

### Bat species identification

Bats were identified through a combination of cave bat survey methods such as live-trapping with mist nets, acoustic analysis using a bat detector (Dodotronic Ultranic 250K, Brazil) and visual surveys. Six meter long mist nets were placed along the entrance and flyways inside the cave. Individual bats were collected and identified by the Zoological and Wildlife Museum of the University of the Philippines Los Baños Museum of Natural History (UPLB-MNH) based from the guide “Key to the Bats of the Philippine Islands” [37]. Bats were immediately released upon identification. No bats were killed in this study.

### Elemental analysis of bat guano

The elemental compositions of bat guano C1 and C2 were analyzed semi-quantitatively using handheld X-Ray Fluorescence Spectrometer (Delta Professional, Olympus, Japan) at the Earth Material Science Laboratory of the National Institute of Geological Science, University of the Philippines Diliman. Guano samples were dried at 105°C and elemental analysis was done in triplicates. Light elements, Silver (Ag), Cadmium (Cd), Tin (Sn) and Antimony (Sb) were removed to normalize the results and reported as semi-quantitative or based on a relative composition.

### DNA extraction, amplicon library construction and sequencing

Total DNA was extracted using the PowerSoil DNA Isolation Kit (MoBio, Solana Beach, CA, USA) and used as template for polymerase chain reaction (PCR) amplification with primer set 341F (5’-CCTACGGGNGGCWGCAG-3’ and 805R: 5’-GACTACHVGGGTATCTAATCC-3’) targeting the V3-V4 region of the 16S rRNA gene. Amplifications were performed in a final volume of 50 μl containing 10X Taq buffer, dNTP mixture, 10 mM of each primer and 2 U of Taq polymerase (ExTaq, Takara, Japan). Cycling conditions in C1000 Touch thermal cycler (Bio-Rad, Hercules, CA, USA) were: initial denaturation at 95°C for 3 min; followed by 25 cycles of 95°C for 30 s, 55°C for 30 s, 72°C for 30 s; with final extension at 72°C for 5 min [38]. The PCR products were confirmed using gel electrophoresis and purified using the Agencourt AMPure XP Reagents beads (Bechman Coulter, Brea, CA, USA). Equal amounts of purified product were pooled, and final product quality were assessed on a Bioanalyser 2100 (Agilent, Palo Alto, CA, USA) using a DNA 7500 chip. Sequencing was performed by Chunlab Inc. (Seoul, Republic of Korea) with Illumina MiSeq 250 paired-end system (Illumina, San Diego, CA, USA), in accordance with the manufacturer’s instructions.

### Bioinformatic and statistical analyses of C1 and C2 guano

Data preprocessing and taxonomic classification of C1 and C2 sequence reads were carried out in Mothur pipeline [39]. Forward and reverse reads, after primer and barcodes removal, were aligned to form one continuous DNA sequence. Sequences that contained ambiguous bases less than 430 bp in length, or with homopolymers of greater than 8 bp were discarded. Chimeras were identified and removed using UCHIME [40]. SILVA reference database release 128 [41] was used for sequence alignments and classification. The microbial diversity was analyzed using QIIME software [42]. In addition, the upstream analyses of microbial sequences through Mothur pipeline were also complemented with QIIME tool.

Alpha diversity analysis included Shannon index, Simpson, Chao1, and observed number of bacterial species. The functional profiles of microbial communities were predicted by the software “Phylogenetic Investigation of Communities by Reconstruction of Unobserved States (PICRUSt)” and Kyoto Encyclopedia of Genes and Genomes (KEGG) Database [43]. The phyloseq, biom, and pheatmap R packages were used for data analysis and plotting. Metagenome sequence data of C1 and C2 guano are available at NCBI with accession no. SRP101645.

### Comparative analysis of bat guano microbiome

C1 and C2 bat guano data were compared with published microbiome profiles retrieved from NCBI SRA (Sequence Read Archives) in fastq or fasta format [22, 35, 44], according to protocol. Briefly, data were quality filtered using Mothur. Good quality sequences, defined as sequences having a length of at least 100 bases, contain homopolymers of not more than 5 bp, and having zero ambiguity were used for comparison. All the filtered sequences were merged into a single fasta file and processed using QIIME pipeline. SILVA123_QIIME_release was used as reference to pick OTUs employing a closed reference approach based from different studies that utilized different regions of the 16S rRNA gene. Non-bacterial OTUs were removed from the dataset. Core OTU was defined as an OTU present in 100% of the samples. Correspondence analysis of the samples was done in R using the FactoMineR package [45].

## Results and discussion

### Cabalyorisa Cave, Mabini, Pangasinan

Mabini is a third-class municipality located in the Western district of the Province of Pangasinan, Philippines. Mabini, named after the sublime paralytic Philippine hero, was once part of the Province of Zambales and formerly known as Balincaguin or “Bali lan Caguin”, a Zambal phrase which translates to “Abode of Bats” [46]. The municipality lies at about 15°55’00” and 16°52’00” longitudes and about 119°56’00” and 120°04’00” latitudinal lines and bounded on the north by the City of Alaminos and town of Sual, on the northwest by the municipalities of Agno and Burgos and on the southwest by the municipality of Dasol [36, 46]. The town was formerly named Balincaguin because of its numerous karstic and limestone caves that include Cacupangan Cave, Binmatya Cave, Ara-saas and Santo Rosario Caves, and Tinmori Tower Karst [47] which serve as roosting sites for various species of bats.

### Bat guano

A combination of several approaches on bat survey, namely, live-trapping with mist nets, acoustic monitoring using bat detector and visual surveys, were used. Bats inside Cabalyorisa Cave were mainly insectivorous and identified as *Miniopterus australis*, little long-fingered bat or little bent-winged bat; *M*. *schreibersii*, a common bent wing bat and *Rhinolophus amplixedectus*, the horse shoe bat. Encinares (2016) documented that aside from macroarthropods, microarthropods observed included isotomid, sminthurid and an unidentified springtail, *Geolaelaps*, *Oplitis*, *Deraiophorus*, two uropodids and one oribatd mite, and an unidentified brown ant. Presence of these microarthropods confirmed the insect-feeding nature of the bats roosting in Cabalyorisa Cave [36].

XRF spectrophotometric analyses of C1 and C2 samples revealed significant differences in the composition of both the major and trace elements (Table 1). C1 guano had high levels of Si, Fe, Mg, Al, Mn, Ti and Cu while C2 samples yielded high concentrations of Ca, P, S, Zn and Cr. The differences may be attributed to the decaying process once the guano were deposited on the ground or on rock crevices. Decomposition of bat guano is affected by various physical and environmental factors [22] and the dynamic nature of the microorganism present. The conversion of organic and inorganic matters by the microorganism present, in turn, results in the change of pH due to the release of ammonia. Thus, the resulting high levels of nutritional contents from major and trace elements are vital components of organic and inorganic compounds used in biosynthesis and energy production supporting the growth and diversification of guano-borne bacteria [48]. Sequentially, high nutrient levels of guano promote the growth of diverse groups of microorganisms.

**Table 1:** Elemental composition of bat guano from Cabalyorisa Cave using XRF spectrophotomer.

	Elements^a^, %											
Sample	SiO_2_	CaO	Fe_3_O_4_	P	S	MgO	Al_2_O_3_	Mn	Zn	Ti	Cu	Cr_2_O_3_
**C1**	26.52	23.63	15.45	6.12	1.66	7.45	13.61	4.72	0.21	0.35	0.23	0.05
**C2**	15.83	38.68	11.51	13.01	4.94	5.68	8.19	1.15	0.40	0.17	0.20	0.25

^a^Legend:

SiO—Silicon monoxide

Al_2_O3—Aluminum oxide

CaO—Calcium oxide

Mn—Manganese

Fe_3_O_4_—Iron oxide

Zn—Zinc

P—Phosphorus

Ti—Titanium

S—Sulfur

Cu—Copper

MgO—Magnesium Oxide

Cr_2_O_3_—Chromium oxide

### Richness and diversity analysis of bacterial community composition from two guano samples

A total of 220,152 and 169,161 reads were obtained from C1 and C2, respectively. After contig assembly, trimming, and chimera removal, a total of 104,764 and 87,379 valid reads were obtained for C1 and C2, respectively. Valid reads for C1 were assigned to 12,435 OTUs, while valid reads for C2 were assigned to 5,408 OTUs.

**Fig 1:**
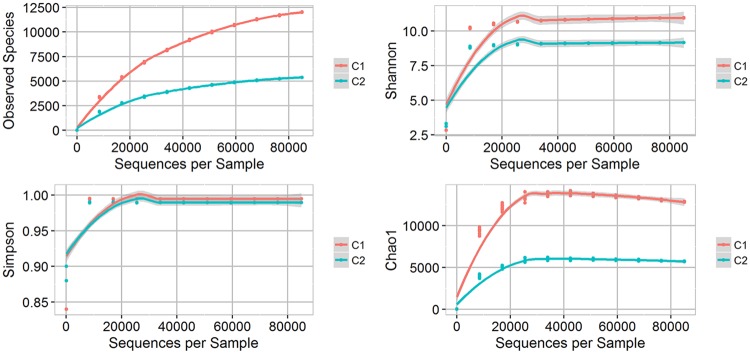
Shannon, Simpson, and Chao1 indices, and the number of observed species in bat guano samples, C1 (red) and C2 (blue).

Rarefaction was done to a maximum depth of 85,000 counts per sample and 10 replicates per iteration. The results of the Shannon, Simpson, and Chao1 indices, and the number of observed species are show apparent variations between the two samples (Fig 1). Analysis of within-sample alpha diversity suggests that the bacterial community of C1 and C2 samples exhibited high biodiversity in all tested metrics, with C1 being more diverse than C2. Comparison of observed species and Chao1 diversity indices suggest adequate sampling of the communities in both samples.

In order to determine the difference in bacterial diversity between two cave locations, the true diversity was computed using Shannon-Weiner Index and Shannon entropy. Since diversity indices alone cannot distinguish two or several unequal samples, it is desirable to determine the number of equally-common genus termed as “effective genus number.” In this study, effective genus number was calculated as a function of the natural exponent raised to a Shannon entropy value, estimated according to Chao and Shen’s formula from the Shannon index [49, 50]. C1 was computed to have an effective genus number of 11.33, while C2 effective genus number was 2.04. Thus, the bacterial diversity at genus level in C1 is five times higher compared to C2, despite yielding lesser number of OTUs.

### Bacterial composition in the two guano samples

**Fig 2:**
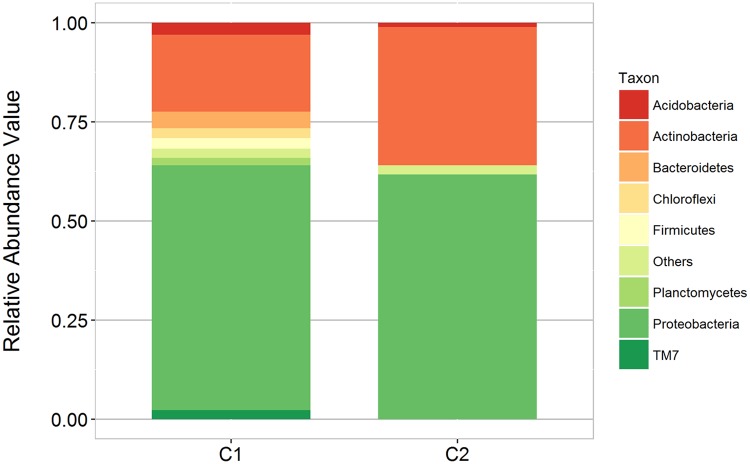
Comparison of the phylum level distribution of bat guano samples, C1 and C2.

Bacterial OTUs of the representative sequences were taxonomically assigned to a total of 30 phyla excluding the unclassified reads. Most OTUs within these phyla belonged to unclassified groups suggesting the presence of novel groups of microorganisms. Apparent dominance of Proteobacteria, Actinobacteria, Bacteroidetes, Firmicutes, Chloroflexi, candidate phylum TM7 (or Saccharibacteria), and Planctomycetes in the C1 and C2 guano samples was observed (Fig 2). C1 and C2 shared 2,467 OTUs (24.8%) classified under 15 phyla, despite being obtained from the same cave system inhabited by the same species of bats. The differences between C1 and C2 microbiome profiles were attributed to these factors: a) the physico-chemical properties of guano; b) decaying process or age of the sampled guanos; c) host species; d) surrounding environment.

**Fig 3:**
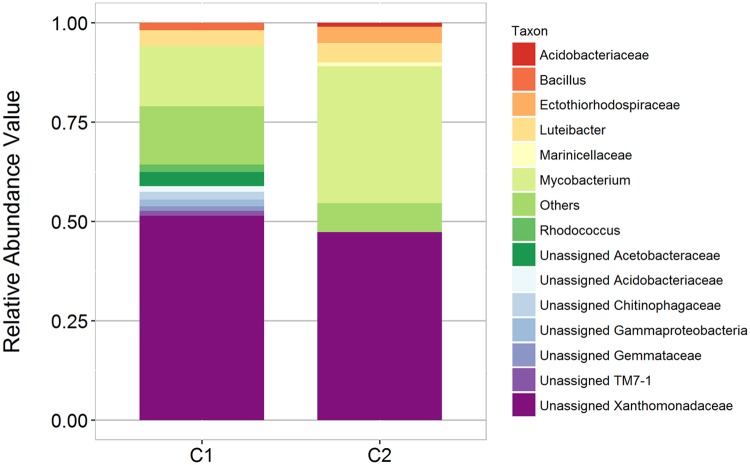
Relative abundance of dominant core bacterial genera present in bat guano samples, C1 and C2.

Core microbiome of C1 and C2 revealed 117 shared genera. Fourteen genera have relative abundance greater than 1% which is dominated by unassigned Xanthomonadaceae and *Mycobacterium* (Fig 3). Other significantly abundant genera on both C1 and C2 samples include *Bacillus* (1.9% and 0.18%), *Luteibacter* (3.8% and 5.2%) and *Rhodococcus* (1.6% and 0.14%). Much of the core microbiome members are yet to be classified.

C1 and C2 samples harbored potentially pathogenic genera such as *Burkholderia*, *Corynebacterium*, *Francisella*, *Legionella*, *Mycobacterium*, *Pseudomonas*, and *Rickettsia*. This suggested that guano may act as source of microorganisms that could potentially be pathogenic to humans and animals [51]. The frequent intra- and inter-roost movements and long distance migrations of bats enhance the potential of bacterial transmission among individuals [17, 22, 25, 52]. Presence of beneficial bacteria in bats like probiotics can provide vital functions to their host such as processing of skin proteins, freeing fatty acids to reduce invasion of transient microorganisms and inhibiting pathogenic microorganisms [53–57]. *Pseudomonas* spp. isolated from bats, amphibians and plants produce antifungal properties particularly against *Pseudogymnoascus destructans*, a causative fungus of white-nose syndrome in bats [57]. Dietary habits were, likewise, reported to affect the bat gut microbiome and allow the acquisition and transmission of infectious agents to other bats and organisms inside a cave. [53–55]. Indeed, several insects consumed by insectivorous bats have been found to harbor harmful bacteria and indigenous bacteria common in human and animal diseases, including *Salmonella*, *Yersinia* or *Campylobacter* species and several enteric pathogens [22, 25, 54, 58–64]. Similarly, contaminated fruits [51] or water [25] might also be possible sources of bacteria.

Many members of identified core microbiome of C1 and C2 were uncultured or unclassified. These data validate possible presence of novel microorganisms found in the guano samples from Cabalyorisa Cave, Mabini, Pangasinan.

### Functional profiles of the two guano samples

**Fig 4:**
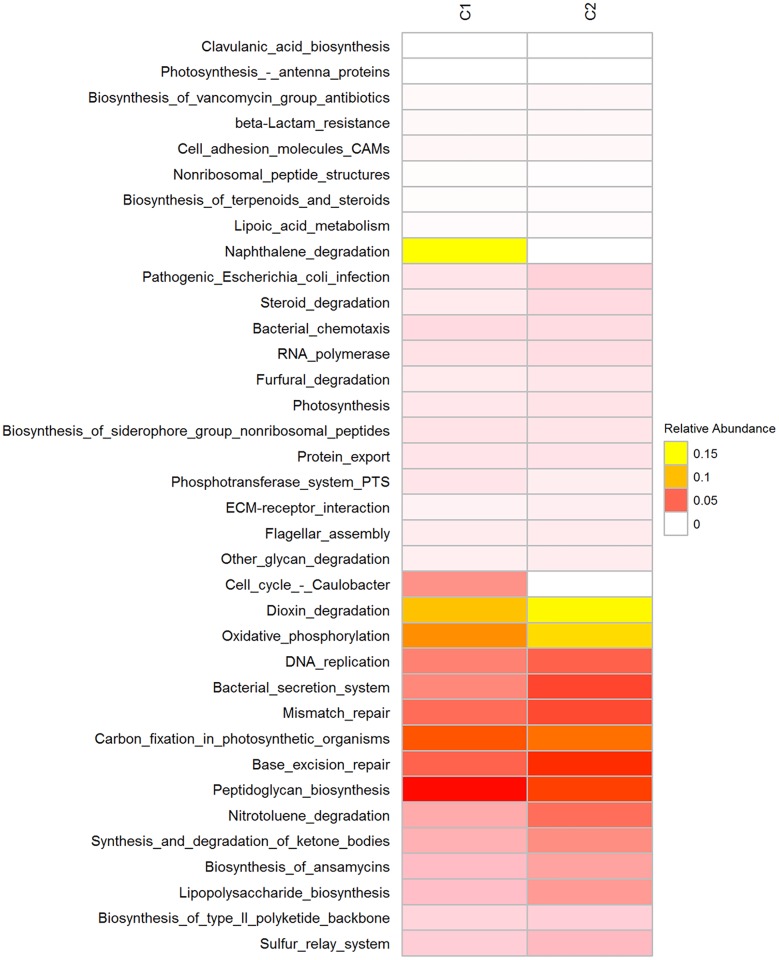
Relative abundance of predicted function of C1 and C2 guano microbiome.

A total of 1136 OTUs were used to predict the functional profile of the microbial communities. Using PICRUSt, genes found to be involved in metabolism of carbohydrates, amino acids, nucleotides, lipids, xenobiotics and other compounds were both equally abundant in C1 and C2. Pathways involved in biosynthesis of other secondary metabolites such as antimicrobials (i.e. streptomycin biosynthesis, novobiocin biosynthesis, stilbenoid, diarylheptanoid and gingerol biosynthesis, penicillin and cephalosporin biosynthesis, etc.) are inferred based on the samples. Other pathways involved in metabolism of xenobiotics and of other compounds (i.e. polycyclic aromatic hydrocarbon degradation, chloroalkane and chloroalkene, naphthalene, benzoate, aminobenzoate degradation, bisphenol, caprolactam, etc.) were also predicted (Fig 4).

Based on predicted functions of C1 and C2 microbiome, the enrichment of genes related to pathways involved in metabolism of carbohydrates, amino acids, lipid, and energy were comparably abundant in C1 and C2. This suggested role of microorganisms active in decay and biogeochemical processes. Moreover, pathways involved in the biosynthesis of secondary metabolites such as antimicrobials and other degrading recalcitrant compounds indicated presence possible novel of microorganisms that produce these metabolites.

### Comparative analysis of bat guano microbiome

**Fig 5:**
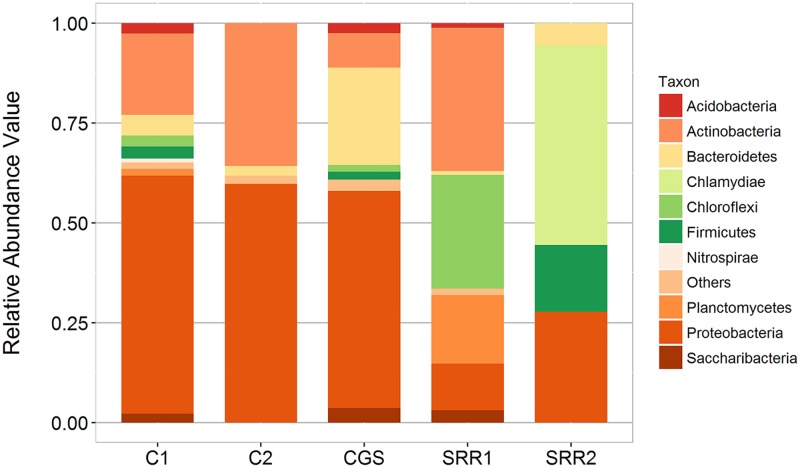
Phylum level distribution of different microbiome studies compared.

Bat guano microbiome have been reported from composite guano collected on the cave floor (no information about the host bats) and on fresh and decaying guano (source bat is *Rousettus eschenaultia*) from Robber’s Cave in India [22]. Analysis of C1 and C2 microbiome data along with previous data from NCBI GenBank database revealed differences in terms of composition and relative proportion of bacterial communities. The relative abundances of major phyla in previous studies were different. Quality filtered and analyzed sequence reads (SRR868695) from the study of Veikkolainen et al. (2014) [44] showed only four dominant phyla, namely, Chlamydiae (50%), Proteobacteria (27.8%), Firmicutes (16.7%), and Bacteroidetes (5.6%). In the SRR1793374 study of de Mandal et al. (2015) [34] and the CGS study of Banskar et al. (2016) [22], at least 28 and 22 bacterial phyla were detected (Fig 5), respectively. Actinobacteria, Chloroflexi, Planctomycetes, and Proteobacteria dominated in the study of de Mandal et al. (2015) [35] while Proteobacteria (54.2%), Bacteroidetes (24.4%) and Actinobacteria (8.6%) were the most dominant phyla in the CGS sample of Banskar et al., (2016) [22].

**Fig 6:**
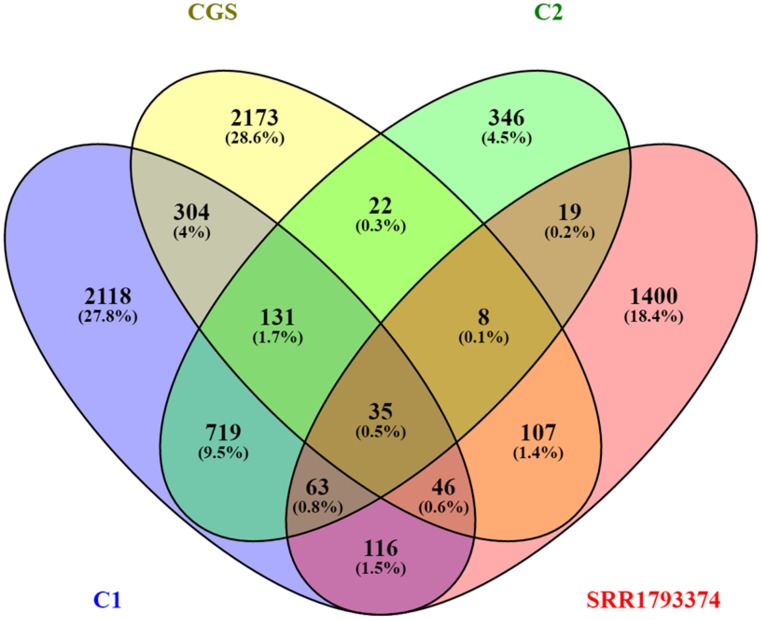
Venn diagram showing the shared OTUs among the different microbiome studies compared.

**Fig 7:**
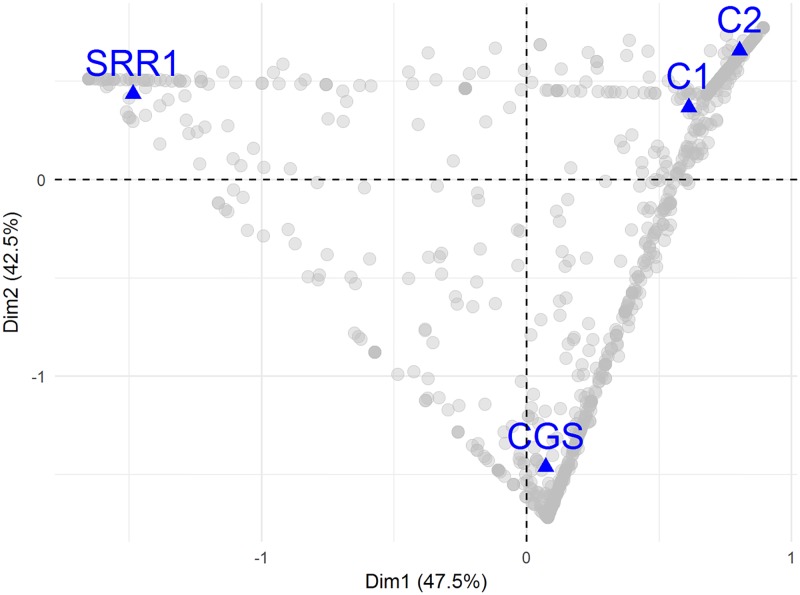
Correspondence analysis map of the decaying guano samples at genus level.

To further compare the samples, beta diversity analysis using CGS sample of Banskar et al., (2016) [22], the composite guano samples of de Mandal et al., (2015) [35] and the C1 and C2 samples were done in QIIME. This comparison was conducted for the representatives of decaying guano samples. The core microbiome of CGS, composite guanos, and C1 and C2 showed 35 core OTUs (Fig 6). At the Phylum level, the major phyla common to all samples are Acidobacteria, Actinobacteria, Chloroflexi, Nitrospirae, Proteobacteria, and Saccharibacteria. Bray-Curtis distance and UPGMA cluster analyses revealed that C1 and C2 guano samples were more similar than the composite guano and CGS sample. This suggested that C1 and C2 microbiome exhibited some unique microbiome profiles, further illustrated in the correspondence map of four guano samples shown in Fig 7. Moreover, CGS and SRR1793374 microbiome profiles were remarkably different from those of C1 and C2.

Across samples, relatively high abundance of Actinobacteria suggested that members of this phylum were conserved in bat guano. Growth of acidophilic Actinobacteria was favored due to nutrient-rich acidic bat guano [65].

**Fig 8:**
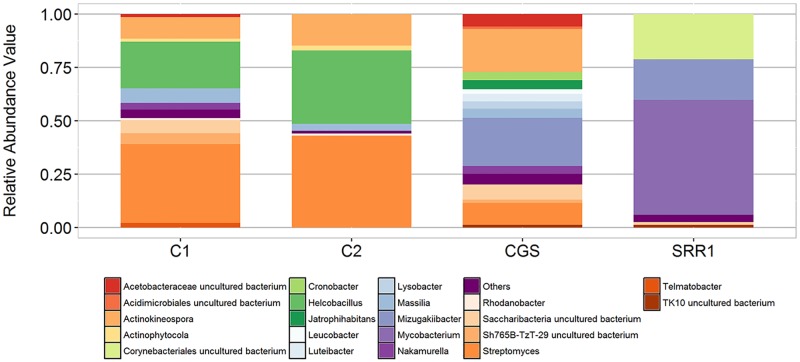
Genus level distribution of core microbiome of different guano samples.

At the genus level, core microbiome of CGS, SRR1793374, C1 and C2 samples shared 27 bacterial genera (Fig 8). Majority of the 27 identified core genera are from Actinobacteria, 12, and from Proteobacteria, 11. Other genera include *Telmatobacter* (from Acidobacteria), uncultured Chloroflexi, *Leptospirillum* (Nitrospirae), and an uncultured Saccharibacteria. Relative abundance of each genus varied across the samples. *Streptomyces* was one of the most abundant genera in the core microbiome, followed by *Mycobacterium* (Fig 8). A number of genera under Actinobacteria isolated from limestone caves, such as *Streptomyces* and *Actinomyces*, were known to produce antimicrobial compounds [66, 67]. Furthermore, some genera in the core microbiome, such as *Mycobacterium*, *Burkholderia* and *Leptospirillum*, were known to have pathogenic species. The observed four microbiomes (including this study) shared common OTU under the Enterobacteriaceae family (Genus: *Cronobacter*). Proteobacteria were overrepresented in C1, C2 and CGS samples while Bacteroidetes were relatively most abundant in CGS [22]. Chloroflexi and Planctomycetes were most abundant in SRR1793374 [35] relative to other samples.

Comparing guano microbiome data of C1 and C2 samples with data on composite and decaying guano revealed that it was more similar to each other and were highly different in microbial composition. Differences among the samples can be explained by origin, host bat species and its diet, duration of the decaying process or age of the guano and other physicochemical properties. In terms of diet, the identified bats (*Miniopterus schebersii*, *M*. *australis* and *Rhinolophus amplixedectus*) were observed to be mainly insectivorous, while *R*. *eschenaultia* from Robber’s Cave is frugivorous. Phillips et al. (2012) reported that herbivorous and reproductively active bats carried more diverse microbiota than carnivorous and reproductively inactive individuals [68]. According to Carillo-Araujo et al. (2015), diet primarily defined the gut microbiome [62]. Banskar et al. (2016) observed that dietary overlap among frugivorous and insectivorous bats explained the similarities in gut microbial communities [25, 48]. Microbiome profiles observed in fresh and decaying guano might have been affected by the age of the guano or by the decaying process dependent on physical and environmental factors [25]. As the guano age, microorganisms initially present in the guano carry out various biogeochemical processes such as organic and inorganic nutrient cycles leading to changes in pH and nutritional contents determining increase in bacterial growth and diversity. This could explain the difference in microbiome of fresh and decaying guano observed by Banskar et al. 2016 [22]. Microorganisms present on the soil or cave floor where the guano, was obtained also contributed to the differences.

## Conclusion

High-throughput sequence-based analysis with Illumina MiSeq was used to describe the first in-depth study on bacterial community profiles of bat guanos collected in a Philippine cave. The microbiome profiles of the Cabalyorisa bat guano exhibited unique characteristics. Metagenomic analysis of OTUs from bat guano revealed it as a source of potentially pathogenic bacteria and/or novel functional genes. Difference on bacterial diversity between C1 and C2 samples may be attributed to differences of host species, diet and other physico-chemical characteristics of the guano and its environmental surroundings. This first report on bat guano collected in Cabalyorisa Cave, Mabini, Pangasinan stirred scientific interest for possibilities of isolating and characterizing novel bacteria with multiple functions for production of antibiotics and enzymes. On the downside, the presence of potential pathogenic bacteria may impose health hazards to local folks harvesting guano for agricultural purposes.
