# Correction: Bacterial diversity of bat guano from Cabalyorisa Cave, Mabini, Pangasinan, Philippines: A first report on the metagenome of Philippine bat guano

**DOI:** 10.1371/journal.pone.0205947

**Published:** 2018-10-11

**Authors:** Marian P. De Leon, Andrew D. Montecillo, Dale S. Pinili, Maria Auxilia T. Siringan, Doo-Sang Park

There is an error in the second sentence under the “Bat guano” heading in the Results and discussion section. The correct sentence is: Bats inside Cabalyorisa Cave were mainly insectivorous and identified as *Miniopterus australis*, little long-fingered bat or little bent-winged bat; *M*. *schreibersii*, a common bent wing bat and *Rhinolophus arcuatus*, an arcuate horseshoe bat.
